# Genetics of Normotension Preventing Hypertension Leads to a Novel Physiological Paradigm

**DOI:** 10.31083/j.rcm2304119

**Published:** 2022-04-01

**Authors:** Alan Y. Deng

**Affiliations:** ^1^Research Centre, Centre hospitalier de l’Université de Montréal, Montréal, QC H2X 0A9, Canada

**Keywords:** genetics of normotension, hypertension suppression, regulatory hierarchy

## Abstract

Possessing blood pressure in normal ranges is considered healthy, and does not warrant medical attention for obvious clinical reasons. However, to realize normotension and then maintain it even when confronted with a hypertensive threat must have its biological ‘shield of armour’. While sensitivity to hypertension has been widely recognized and studied, inherent mechanisms that enable a physiological resistance to hypertension to occur have received little attention. Recent advances in normotension genetics have produced unexpected insights. A hypertension ‘suppressor’ likely inhabits the normotensive genome of inbred Lewis rats. This suppressor behaves as a ‘master’ control capable of functionally abrogating the effects of hypertension-promoting alleles from multiple quantitative trait loci. This conceptual advancement lays the foundation for uncovering an anti-hypertension gene. Discovering its identity will assist our attempts at developing innovative diagnostic and therapeutic strategies for circumventing and treating hypertension. This new domain of suppressing hypertension goes beyond the conventional pharmacological treatments of hypertension before symptoms appear. For this purpose, a valid theoretical basis and framework is needed that can interpret the experimental data and produce testable predictions for authenticating, enriching or amending the normotension paradigm in the future.

## 1. Introduction

### 1.1 General Background

Hypertension happens to about 30% of general populations and chronically elevates the risk for debilitating cardiovascular and renal diseases and stroke [1]. Because of these health ramifications, much scientific research has focused on uncovering causes of human hypertension in both polygenic [2, 3] and monogenic forms [4, 5]. Greatly under-stressed or even heeded is the fact that 2 out of 3 people world-wide preserve their blood pressure (BP) in normal ranges, and some people never develop hypertension, despite adverse effects. Some forms of normotension are resistant to certain hypertensive impact [6]. Thus, a lack of hypertension susceptibility does not necessarily establish normotension. Normotension formation or ‘Normotensionogenesis’ is not merely a mirror image of the hypertension pathogenesis, despite often phenotypically appearing so. A physiological antidote against hypertension must innately persist in the form of normotension in general populations.

### 1.2 Need to Study Normotension

Evolutionarily, pressures to fitness by natural selection [6] should favour normotension. Detrimental effects from mutations causing hypertension need to be lessened biologically by having intricate function networks that robustly stabilize the BP homeostasis in defiance of actions from susceptibility genes and environmental fluctuations [7]. Otherwise, hypertension would be more prevalent than normotension, instead of the inverse. Notwithstanding this acquiescence, until recently very little vigorous research has been undertaken to decipher fundamental mechanisms of attaining and sustaining normotension that concomitantly opposes hypertension [8, 9, 10].

### 1.3 Questions Concerning Normotension

The appreciation entails some questions: (a) Is normotension valuable solely as a mere opposite control tool for hypertension susceptibility, or does it have distinct etiologies in its own right? (b) What are the mechanisms that allow the majority of people to sustain normotension? (c) Can normotension reveal the BP control mechanisms that counter hypertension? Elucidating preservative mechanisms of gene alleles against hypertension, and on the basis of it, repairing or restoring protective functions in individuals lacking them can contribute to our endeavors at reducing the occurrence of hypertension. Since normotension not approaching hypotension is beneficial to our health with no discernible physiological consequences [1], pharmaceutically enhancing it or a gene therapy targeting it as an anti-hypertension measure is not likely to compromise the integrity of cardiovascular system.

### 1.4 Known Blood Pressure Regulations

Many living land mammals including rodents and humans attain a similar level of blood pressure. Their averaged systolic blood pressures are in 120 s–130 s mmHg [11]. Mechanically, mammalian blood pressure is controlled by cardiac output, total peripheral resistance and arterial stiffness [12]. Its homeostasis is integrally modulated by networks of renal, neuronal, humoral, cardiovascular, vasoactive, and hormonal actions [13, 14]. The most well-known vasoactive action is via the renin-angiotensin-aldosterone system (RAAS) [15]. Recently, immunity [16] and gut micro-organisms [17] were found to be associated with BP.

### 1.5 Review Outlines

In this review, (a) the classical hypertension genetics paradigm will be revisited. (b) Certain retrospective phenomena pointing towards hypertension-prohibiting normotension will be ascribed and transpired into verifiable hypotheses. (c) Recent experimental evidence will be gathered to form a paradigm governing ‘normotensinogenesis’ opposing hypertension. (d) Confirmable predictions founded on the paradigm will be offered and strategies to test them proposed. (e) The paradigm will be interfaced with the hypertension susceptibility to build a unified physiological foundation for the BP homeostasis and therapeutic applications.

## 2. Generic Paradigm for Controlling Blood Pressure as a Polygenic Trait

### 2.1 Purported Genetic Prototype for BP Determination

**Fig. 1.**
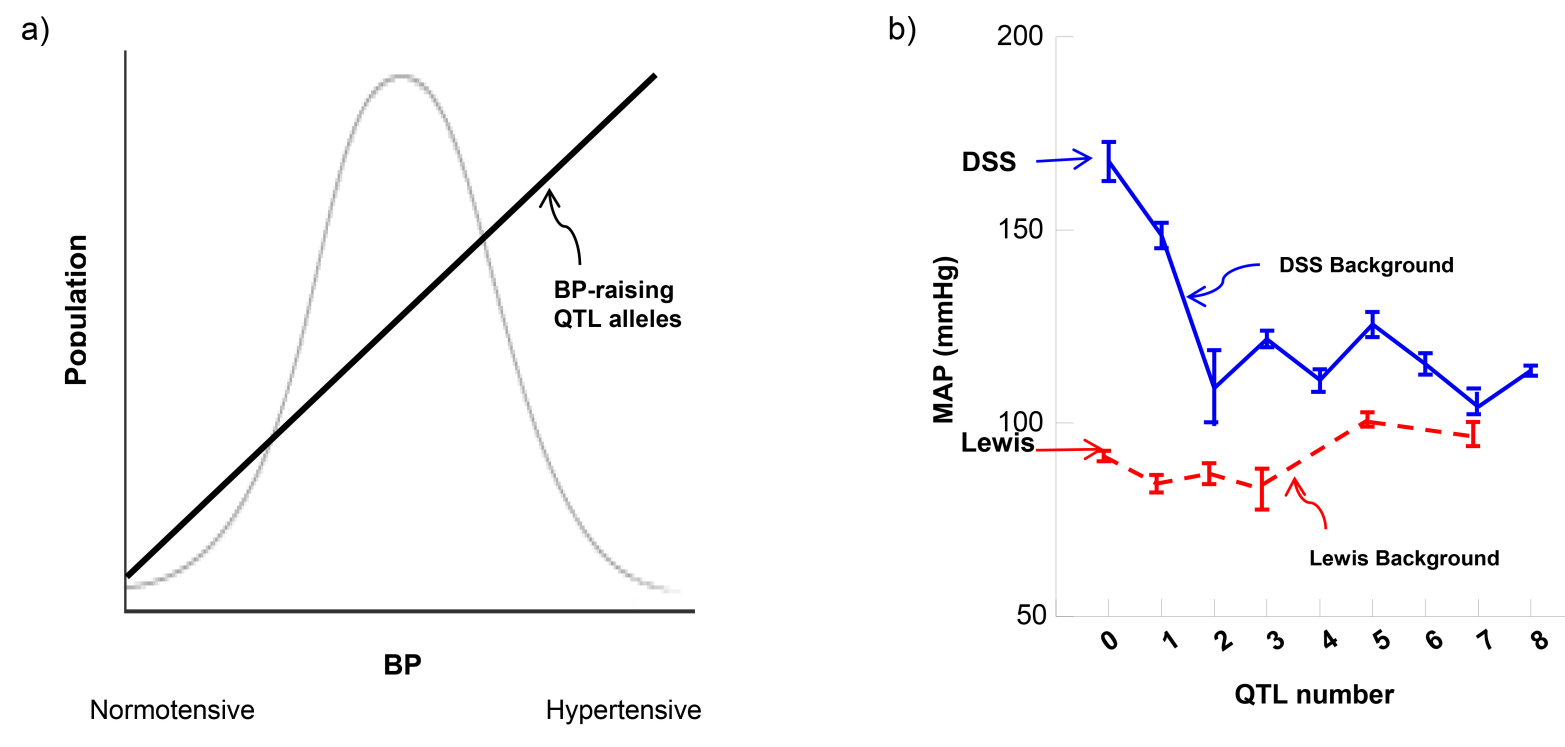
**BP determination: theory versus reality**. (a) BP distribution and a theoretical quantitative genetic basis explaining it. (b) Non-linear correlation in biological function leads to the modularity paradigm on the DSS background [19]. Non-numerical correlation on the Lewis background [8] disapproves the quantitative genetics assumption [18] that normotension is determined biologically by accumulative aftereffects from multiple BP-diminishing QTL alleles. Dahl salt-sensitive (DSS) and Lewis rats provide the baseline and reference BP values at two extremes (right-pointing arrows) where no QTLs are introgressed. 1 to 8 correspond to the number of QTLs of BP-increasing or decreasing input respectively. QTL, quantitative trait locus.

Principled as a quantitative trait [18], BP is distributed in populations as a bell curve with hypertension and normotension at 2 extremes (Fig. 1a, Ref. [8, 18, 19]). Genetically, a point on the curve is assumed to be attained by a varying degree of quantitative trait loci (QTLs) present. Quantitative genetics [18] provides an estimate on the influence of each locus. A QTL refers to a locus residing in a chromosome segment in genetic terms, but a QTL is a single gene when molecularly identified. For example, *C17QTL1* on rat Chromosome 17 is a single *Chrm3* gene encoding [muscarinic cholinergic receptor 3 (M3R)] [20, 21, 22, 23]. No combination with another gene is necessary to affect blood pressure.

### 2.2 Predictions and Support

Based on this postulate, several predictions are apparent. Multiple QTLs with varying effects are expected to be mostly additive [2, 3], thus modifying the BP phenotype and moving them cumulatively along the bell curve. Multiplying BP-raising QTL alleles would propel BP towards hypertension; conversely, fewer BP-raising alleles would provoke a normotensive shift. Hypertension and normotension would simply represent two extremities in a continuum on a BP variation curve. Thus, identifying a hypertensive QTL allele would yield a mechanism impairing the normal BP homeostasis, and opposite is self-explanatory for achieving normotension.

Some empirical evidence seems to support certain aspects of these predications. By selective inbreeding, some hypertensive and their normotensive rodent strains [24, 25] have been established, e.g., hypertensive Dahl salt-sensitive (DSS) rats and normotensive Lewis rats. They appear to inherit BP bimodally with no overlap between them (Fig. 1). Although DSS is known to develop hypertension in a salt-accelerated fashion, its use as a model for generic hypertension on low salt is evident. For example, DSS-based *Chrm3* nulls lower blood pressure under both low and high salt diet [20]. QTLs from DSS as a functional proxy have captured QTL orthologs [26, 27] from humans in general populations under normal salt diets [2].

## 3. Genetics of Hypertension Susceptibility

Efforts at unraveling molecular bases of polygenic hypertension have concentrated on susceptibilities to BP. Genome-wide association studies (GWAS) in humans have statistically localized hundreds of BP QTLs [2, 3]. Studies using inbred animal model have found similar results [25, 28]. Physiological roles of these QTLs together in controlling overall BP turn out to be in modularity in both humans and animal models [19, 26, 27]. Modularity means that certain QTLs function as a group as if only one QTL existed, and their combined effects on BP is non-cumulative [29]. This subject will be discussed further in the future research.

##  4. Appearance of Anti-hypertension Normotension

### 4.1 Conventional Views on Achieving Normotension

In all the above studies, normotensive subjects were no more than controls for their hypertensive counterparts [2, 3]. An intuitive assumption based on quantitative genetics [18] is that mirroring in mechanisms yet opposite in effects to hypertension, normotension may be acquired in a computable fashion from BP QTLs, i.e., via a mathematical reverse of hypertension. However, some experimental data questioned this arithmetic premise.

### 4.2 Distinct Normotensive Backgrounds Affecting BP QTL Detections Differently from Hypertensive Backgrounds

Normotensive backgrounds of Dahl salt-resistant rats (DSR) [30, 31], Lewis [8, 32, 33], Sabra hypertension-resistant (SBN/y) [34] and possibly Brown Norway (BN) [35] rats do not allow BP to vary with BP-elevating QTL alleles, even from multiple QTLs. Among modifiers affecting expressions of genes [36], protective genes seem to be a special class such as those influencing type 1 diabetes [37]. Some normotensive strains are hypertension-permissive such as DSR [38], Wista Kyoto (WKY) [39] and Milan normotensive (MNS) rats [40].

Is the hypertension resistance owing to an insufficient quantity of QTL alleles that raise BP in a normotensive strain such as Lewis, or due to the existence of a hypertension ‘suppressor’? Epistasis and pleiotropy [18] could also be involved.

## 5. Genetics of Normotension Countering Hypertension

### 5.1 Experimental Evidence

Normotension of the inbred Lewis strain [30, 31] was used for genetic analyses. Since BP-altering QTL alleles have no effect on BP in the Lewis background, whereas the same QTL alleles changed BP in the hypertensive DSS background in reciprocal crosses [32], the background seems to either reject or allow BP to exhibit.

A simple test is whether or not Lewis can be made hypertensive by incrementally adding BP-elevating QTL alleles from DSS and simultaneously reducing its BP-decreasing alleles. If the cumulative assumption [18] would hold true, one would expect BP to increase in proportion to the number of contributing BP-raising QTL alleles (Fig. 1a); if not, then, other mechanisms would need to be explored.

Fig. 1b illustrates the actual experimental data [8]. By progressively adding the number of hypertensive DSS QTL alleles in the resistant Lewis background, BP minimally increased, but was not linearly correlated with the quantity of hypertensive alleles added (up to 7 QTLs). Thus, incrementally combining multiple hypertensive QTL alleles by itself does not proportionally drive BP changes and cannot overcome mechanisms of the Lewis genome that resist the rise in BP. The non-effect results are consistent with those of congenic combinations made in the normotensive BN background [35]. A caveat in the BN case [35] is that no reciprocal congenics were made to show that a QTL was involved.

### 5.2 Normotension Is Not because of a Lack of Hypertension

The non-cumulative nature of QTL actions can be biologically validated in the reverse fashion [29]. There is no quantitative correlation between the number of QTLs and BP (Fig. 1b) by incrementally introgressing multiple BP-lowering QTL alleles [19] in the DSS background [32].

Thus, the Lewis normotension is not a result of lacking an adequate number of BP-diminishing QTL alleles in the genome. If DSS’s hypertension proclivity can be viewed as results of pathogeneses caused by the QTL ‘mutant’ alleles, an absence of it should not be responsible for Lewis’ resistance to hypertension. Something else in the Lewis genome appears to actively suppress the combined hypertensive actions from multiple BP-raising QTL alleles.

### 5.3 Suppressing Hypertension to Achieve Normotension.

The heterozygous DSS/Lewis rats had the same BP as that of Lewis [8], indicating that the Lewis genome is completely dominant over that of DSS. Thus, a resistant element(s) seems to exist in the Lewis genome that inhibits the rise in BP caused by DSS BP-raising alleles. The presence of a hypertension stimulator in DSS, instead of an inhibitor in Lewis, is not consistent with the dominance of the Lewis genome and thus can not be a viable alternative.

Without knowing its exact genome location, a logical strategy toward identifying a hypertension ‘suppressor’ would be to remove it from the Lewis genome and consequently induce a rise in BP. Indeed, by progressively trimming the resistant genome in consecutive backcrosses (BC) [8], a BP increase was achieved by BC3, but not before. A region on Chromosome 18 homozygous for DSS associated with a BP rise was identified. This suggests a potential hypertension ‘suppressor’ in that Lewis region [8]. To my knowledge, the only published example of a similar scenario is a suppressor that protects against lupus in mice [41].

## 6. Conceptualizing Hypertension Suppression

The suppression phenomenon signifies no influence whatsoever on BP, and is not the same as the classical epistasis, which refers to one QTL masking a notable outcome of another on BP [10, 42]. This distinction implies a mechanistic separation, because epistasis between 2 QTLs can show a regulatory hierarchy between them only when the suppression is absent [10, 19]. In its presence, no effect from any single QTL can even be shown [8, 9], let alone exhibit an epistatic hierarchy between 2 of them.

## 7. A Contra-intuitive Concept for a Novel Paradigm, i.e., Hypertension Suppression

### 7.1 Hypertension Suppressor as a Master Regulator

A hypertension suppressor acts as a ‘master’ regulator and can nullify cumulative effects of hypertensive QTL alleles [8, 9]. Returning to the questions posed in the introduction, it appears that there is, at least, 2 faces to the BP homeostasis, the hypertension suppression disguised as normotension and hypertension predilection. The propensity to hypertension driven by pathogenic forces can be annulled by the suppressive power. This may be the foundation for new diagnostic and pharmaceutical preventions against hypertension.

At a more fundamental level, the instinctive assumption based on quantitative genetics [18] needs to be broadened and modified to include a role of non-cumulative [29] and hierarchical genetic determination of BP (Fig. 1b).

### 7.2 Other Examples of Master Regulators

Some examples of natural defences against diseases occur in the immune system, mostly behaving in recessive modes [43]. A well-known case is the resistance to infections caused by the human immunodeficiency virus (HIV) [44]. Another example is the cancer resistance that possesses multiple copies of a cancer suppressor [45]. A few instances of a ‘master’ regulator controlling disease resistance have been found in plants [46].

The classical genetics combined with innovative maneuvers [8] has yielded the unforeseen mechanistic insight into hypertension suppression from animal models [8, 9]. No amount of sequencing or using large-scaled statistical methods could have achieved a comparable mechanistic understanding. Without screening by mutagenesis for suppression alleles [47], the Lewis normotension provides a naturally-occurring platform for identifying a hypertension suppressor.

## 8. Rodents and Humans Share Conserved Physiological Mechanisms in Regulating Blood Pressure

### 8.1 An Evolution Conservation

The validity of rodents serving as the effective functional proxy for humans can be seen from the followings.

Rodents and humans diverged around 90 million years ago to become 2 different orders of mammals (www.timetree.org). Obviously, they differ in many aspects of biology, such as size, longevity etc. In spite of these differences, their blood pressures are similar, namely their averaged systolic blood pressures are in 120 s–130 s mmHg [11]. In fact, the majority of living land mammals, except for elephants and giraffes, have similar blood pressures. The environmental impact cannot explain this similarity, because their divergence occurred under 2 drastically different environmental conditions.

The only way to explain this is that basic mechanisms regulating the blood pressure homeostasis must have been established before 90 million years ago in the common ancestor of rodents and primates, prior to the divergence of rodents from primates, and remained little changed up to primates. At that time, no humans existed, but the BP controlling mechanisms were fixed. During the primate evolution, modern humans surfaced only about 300,000 years ago, and acquired the same mechanisms in tandem as rodents up to the present day. This conserved usage of the same BP-regulating mechanisms happened despite that humans gained some extra genome content such as non-coding SNPs (https://www.fortunejournals.com/articles/animal-model-studies-reveal-that-common-humancentric-noncoding-variants-from-epidemiology-are-byproducts-of-primate-evolution-unre.html). That is independently of BP regulating mechanisms [26, 27].

Thus, studying BP regulating mechanisms in rodents is equivalent to studying the same mechanisms in humans. What one sees in rodents reflects the same fundamental mechanisms for humans originating from their common ancestors. Genetic bases and pathways leading to blood pressure regulation should be conserved between rodents and humans.

Then, one may contend, if that is the case, why not directly studying humans, rodents are indirect proxies? This is because we cannot ethically experiment with humans as we can with rodents, such as designed inbreeding, knock out, and knock in. Because of these limitations, we lack functional insights into BP regulating mechanisms by function. As it turns out, the human blood pressure physiology by function is not much different from that of rodents. Nor is it more complicated than that of rodents. It’s simply more difficult to dissect and distinguish than that of inbred rodents due to experimental limitations on revealing the physiology and mechanisms by function.

### 8.2 QTL Modularity/Pathway Is Conserved between Rodents and Humans

When you accept the validity of rodent functional proxy for humans, you may still argue that common mechanisms for blood pressure control have to be demonstrated beyond a global similarity in blood pressure. Indeed, there is evidence that not only individual QTLs, but QTL modularity, are evolutionarily conserved by function between differing orders of mammals such as rodents and humans in controlling blood pressure [26, 27, 48]. By inference, mechanisms in suppressing hypertension originating from their common ancestors are likely to be conserved between humans and rodents

## 9. Future Research Directions

The hypertension-suppression concept creates testable hypotheses that can be experimentally validated and revised.

### 9.1 Capturing a Hypertension Suppressor

The provisional identification of a hypertension suppressor was achieved using limited number of backcross rats [8]. Several genetic experiments can confirm, narrow or even rectify its location or detect new ones. These approaches include a replication with a larger number of backcross (BC) rats, analyzing the rats of intercrosses from the BC2 rats, and producing a congenic strain targeting the suspected segment. Since the hypertension ‘suppressor’ can be pleiotropic, i.e., suppressing the hypertension susceptibility and being a BP QTL in itself, or can be a hypertension suppressor alone, the congenic approach may need hypertension-prone QTL alleles as ‘baits’ to demonstrate a BP-effect.

### 9.2 Mechanistic Implications for Hypertension Suppression

The nature of the hypertension ‘suppressor’ appears to be a substance that the normotensive Lewis rats functionally possess, whereas the hypertensive DSS rats have lost, because the Lewis genome is completely dominant in Lewis/DSS heterozygotes [8]. A threshold including haplosufficiency [49] is another possible mechanism for dominance. In contrast to the hypertension suppressor, the resistant HIV individuals lack a functional chemokine receptor [44] and acts recessively in heterozygotes.

### 9.3 RAAS Impact

In 1 single occasion, the Lewis genetic resistance to hypertension appeared overcome by introducing over-expressed mouse *Renin* genes in a mRen2.Lew congenic strain [50]. Several possible reasons might explain this outcome. First, since the renin-angiotensin-aldosterone system (RAAS) is such a strong force in BP regulations and BP-independent functions [1], an abundant renin might overpower the Lewis hypertension resistance. Second, in mRen2.Lew [50], multiple *Renin *copies were randomly inserted into several genome locations. 1 of the sites could be near or in the hypertension suppressor and consequently have deactivated it. Finally, renal functions in mRen2.Lew were severely undermined and could hinder the effect of the hypertension resistance, as renal cross-transplantations have shown [51].

However, the above explanations could not justify the Lewis resistance to BP-raising QTL alleles from DSS shown in Fig. 1b, since DSS rats have low-renin hypertension [38] and DSS alleles of other RAAS components carried by congenic strains have no effects [8]. Furthermore, none of RAAS genes resides in the Chromosome 18 region where the hypertension suppressor was provisionally located [8], and none exhibits genetic variations between DSS and Lewis [33, 52, 53] as an etiological gene should. Thus, the Lewis resistance to the impact of BP-increasing QTL alleles is indisputable.

Detailed discussions are presented elsewhere [10] on how we may understand ‘disconnections’ between polygenic etiology genes with genetic differences causing BP variations, and BP physiology genes such as *Renin* as well as monogenic hypertension genes.

### 9.4 Research Design in Identifying a Hypertension Suppressor

Unbiased and hypothesis-free genetic approaches will be used in the molecular identification and mechanistic understanding of the hypertension suppressor [8]. Since its power is such that the functional influences from singular and multiple hypertension-prone QTL alleles can be neutralized, its identification has to be done in its absence and following BP changes *in vivo * [10].

Following principles outlined above, a critical requirement is to unequivocally establish an initial chromosome segment(s) carrying the suppressor(s). Afterwards, one needs to limit the number of candidate genes by progressively reducing the size of the suppressor-residing segment. Our recent identification of 3 QTLs is an example [20]. In the process, more than 1 suppressor may appear. The gene responsible for the suppressor must be genetically or epigenetically different between DSS and Lewis. Readily-available rat genome sequences of DSS and Lewis [52, 53] will facilitate this research.

### 9.5 Methodological Approaches

Eventually, the suppressor gene can be molecularly identified by several genetic methods [54]. First, transgenesis of the Lewis suppressor gene into the hypertension-susceptible DSS rat is expected to reduce BP to the level similar to that of Lewis. Second, knocking out the suppressor from the congenic rats made in the Lewis background [8] that carry multiple hypertension-prone QTL alleles from 2 epistatic modules [19] will likely increase BP to the degree close to that of DSS, provided that depleting the suppressor is not embryonic-lethal. Finally, if more than 1 suppressors are involved, the molecular identification approaches will adapt accordingly.

### 9.6 Physiological Significance of Suppressing Hypertension in Blood Pressure Regulations

Beyond the clinical applications, the very existence of a hypertension ‘suppressor’ physiologically reaffirms the deterministic paradigm that a regulatory hierarchy is essential in actualizing the function modality of QTL actions in an organism [10, 29]. It is deducible that the ‘suppressor’ should stand even higher in the regulatory totem pole [10] than the modularized pathways performed by QTL products, because in its presence, BP-raising QTL alleles combined from 2 modules can only modestly increase BP [8].

An inference can be deduced for physiological functions of BP QTLs. Since more QTL components are organized in a sequence analogous to a metabolic pathway [19], the protein product of each QTL within the sequence may not ‘directly’ and immediately affect BP, but rather participates in one step in a cascade of sequential reactions that are distant from the end-phase physiology of the BP homeostasis [29]. Consequently, a defective protein product encoded by a mutated QTL allele can lead to a deficient pathway in regulating BP, and does not necessarily have to act, by itself, as a physiological agent directly impacting BP [10].

The ‘suppressor’ not only prevents hypertensive QTL alleles from elevating BP, but also stabilizes the base-line BP in Lewis rats from dipping, thus constituting a homeostatic buffering capacity [32]. Thus, its function is likely either pleiotropic or an additional BP-stabilizer might be present. In preventing severe hypotension, the ‘stabilizer’ helps guarantee the supply of enough oxygen for normal functions in vital organs.

### 9.7 Studying Other Models of Normotension

The normotensive BN [35] and SBN/y [34] rats appear hypertension-resistant. Studies similar to those reported for Lewis [8] can be performed in these rats to expand, solidify or discover new type of the hypertension suppression. Since not all forms of normotension are hypertension-resistant such as WKY [39] and MNS [40] and possibly DSR rats [38], QTL allele combinations from congenic strains can be carried out using them in reciprocal crosses with appropriate hypertension-susceptible strains. In so doing, one can retest the accumulation-of-the-small effect dogma on QTL actions that are the limited in rat strains (Fig. 1b).

### 9.8 Hypertension Suppression in Reference to Hypertension Susceptibility

The medical significance in the genetics of hypertension susceptibility should not be underestimated [5]. Daunting challenges remain that include identifying a human QTL by function beyond statistics and molecular mechanisms of hypertension-prone QTL alleles, and unraveling the regulatory hierarchies [26, 27]. These tasks have been extensively discussed elsewhere [5, 10, 34].

In the current context of the normotension paradigm, when hypertension suppression [8] is removed as in DSS rats, pathways of QTLs can modularly alter BP [19]. The components in each pathway, their order and regulatory relationships hinge on their epistatic hierarchies and need to be elucidated [10, 29]. Even with the simple genetic evidence, certain QTLs demonstrating opposing BP effects are likely negative regulators of the QTLs downstream in the same pathway [10, 29]. These predictions can and have to be experimentally tested and validated. Other evidence suggests that a post-translational modification may be a basis for a regulatory relationship between 2 BP QTLs exhibiting a similar magnitude of BP effects in the same pathway [53].

### 9.9 Normtension Paradigm in Advancing Human Epidemiology of GWAS 

These physiological insights will rationally accelerate and energize the impending advancements from probabilistic detections of genome locations to function-based mechanisms of QTL actions in the human polygenic BP research [26, 27].

The state-of-the-art GWASs with ever increasing population sizes have enlarged the signal base to hundreds [2, 3]. Aside from the issues of ‘missing heritability’ and a diminishing return in explaining the variance [5], from a functional viewpoint, how are products of the already-identified gene candidates for BP QTLs translated into physiological mechanisms of BP modulations individually and collectively? As shown in Fig. 1b, BP variations are not determined physiologically in proportion to the number of QTL alleles even discounting the hypertension suppression. One-gene-to-1-BP- effect mechanism is less applicable in the polygenic form than the monogenic forms [35]. Pathways involving multiple steps with regulatory hierarchies within and among them appear more plausible in collectively achieving these polygenic functions [10, 29]. Thus, understanding how one QTL acts with another is essential, even with their molecular identity at hand, in the context of a pathway/cascade eventually leading to BP regulation.

### 9.10 Hypertenion Suppression in Personalized Medicine

An obvious therapeutic beneficiary of procuring a targetable hypertension suppressor will be the individuals, either male or female, who are categorized to be hypertension-prone without it, but not yet hypertension-symptomatic. To accomplish this task, identifying and then utilizing a hypertension suppressor will have to coincide with the diagnosticability of hypertension-susceptibility genes that defines a treatable individual. Thus, understanding one molecular mechanism will undoubtedly harmonize and expedite the other for this personalized medicine.

Since normotension bears an element of hypertension suppression, should the opposite be true? In hypertension, can there be a separate hypertension enabler that can facilitate the effects of hypertension-predisposition QTL alleles? If proven valid, a new category of treatment could be added into the anti-hypertension repertoire.

### 9.11 Evolutionary Role of Hypertension

From an evolutionary stance [7], why was hypertension selected in human populations and remains to the present day? One possibility is that in early stages of human evolution, some hypertensive components could have bettered our chances of reproduction and survival. They only become perilous when biological, social, economic, environmental and medical factors have changed such as prolonged life expectancy, life style modifications, improved nutritions, and reductions in various life-threatening diseases. The ‘sickle cell trait’ in heterozygous individuals is a classic example of positive selection in conceding a less serious and age-delayed condition to fight against a more deadly infectious disease that is often fatal in the young, i.e., malaria [55].

### 9.12 Conclusions

Upgrading the prominence and the benefit of normotension capable of resisting hypertension is timely in scientifically and clinically coordinating the gain in genetically dissecting hypertension liability. Comprehending normotension via hypertension suppression will facilitate our overall pursuit in understanding fundamental physiological mechanisms in the BP homeostasis, and provides a conceptual infrastructure and predictions that will open up, reinvigorate and integrate the field of BP genetics, and our fragmented as well as incomplete understanding of BP biology.
